# The efficacy of bleomycin sclerotherapy in the treatment of lymphatic malformations: a review and meta-analysis

**DOI:** 10.1016/j.bjorl.2023.101285

**Published:** 2023-06-29

**Authors:** Jiali Sun, Changfeng Wang, Jing Li, Dan Song, Lei Guo

**Affiliations:** a Children's Hospital Affiliated to Shandong University, Department of Vascular anomalies and Interventional Radiology, Shandong, China; b Jinan Children's Hospital, Department of Vascular Anomalies and Interventional Radiology, Jinan, China; c Shandong Provincial Clinical Research Center for Children's Health and Disease, Shandong, China

**Keywords:** Bleomycin, Lymphatic malformations, Sclerotherapy, Meta-analysis, Efficacy

## Abstract

**Objective:**

At present, bleomycin has been widely used in the treatment of Lymphatic Malformations (LMs). This study aims to perform a meta-analysis to investigate the effectiveness and influencing factors of bleomycin in the treatment of LMs.

**Methods:**

We conducted a systematic review and meta-analysis to clarify the relationship between bleomycin and LMs. PubMed, ISI Web of Science and MEDLINE were searched.

**Results:**

A total of 21 studies (including 428 cases) about bleomycin sclerotherapy for LMs were included in the current meta-analyses. We calculated pooled effective rate and 95% Confidence Interval (95% CI) using random effects model to evaluate the relations between bleomycin and LMs. The results suggested that the effective rate of bleomycin was the combined effective rate was 84.0% (95% CI 0.81‒0.87) and ranged from 39% (95% CI 0.22‒0.56) to 94% (95% CI 0.87–1.02). The heterogeneity among the studies was substantial (I^2^ = 61.7%, *p* =  0.000). In subgroup analyses, it was observed that among retrospective study and prospective study, the estimated effective rate was 80.0% (95% CI 0.76‒0.84) and 91.0% (95% CI 0.85‒0.97), respectively. In terms of the dosage, the combined effective rates of weight-based group and fixed-dose group were 86% (95% CI 0.83‒0.90) and 74.0% (95% CI 0.66‒0.82), respectively. There was no significant publication bias in Egger's test (*p* = 0.059, 95% CI −3.81 to 0.082), but Begg's test did (*p* = 0.023), and the funnel plot is asymmetric.

**Conclusion:**

Our study suggested that bleomycin was safe and effective in the treatment of LMs and was primarily dose dependent.

## Highlights

• Bleomycin was highly effective in treating LMs.
• The usage will affect the efficiency of bleomycin.
• The dosage will affect the efficiency of bleomycin.
• The efficacy of bleomycin was related to classification.

## Introduction

Lymphatic Malformations (LMs), also known as lymphangioma, was previously called cystic hydroglioma,^1^, ^2^ which is a kind of lymphatic malformation and not a malignant tumor. ISSVA also called it low-flow vascular malformation of the lymphatic system).^3^ Existing studies have shown that the incidence of LMs is approximately 1 in 6000 to 1 in 16,000^4^; and can occur in various parts of the body, such as the orbit, armpit, thorax, retroperitoneum, groin, especially in the head and neck.^5^ LMs of the head and neck, when combined with bleeding or infection, can rapidly increase the lumen, leading to disfiguredness, dysphagia, speech problems, and even suffocation, which can be life-threatening.^6^

Bleomycin, an anticancer drug extracted from Streptomyces verticillus,^7^ is cytotoxic, capable of breaking the double strands of DNA and inhibiting DNA synthesis.^8^, ^9^ Bleomycin has been used in a variety of marketers working, Hodgkin’s lymphoma, testicular, ovarian and cervical working.^7^, ^10^ Previous studies have shown that bleomycin can induce cell apoptosis and have the effect of prevent and improve blood vessel damage.^11^ It has become one of the most widely used sclerotherapy for LMs.^12^

Currently, there is no uniform and ideal management to treat LMs.^13^ But there is still no literature about its effectiveness and influencing factors of report, therefore, by reviewing published related research results, we used meta-analysis to verify the efficacy of bleomycin in the treatment of LMs and the influencing factors for the first time.

## Methods

### Literature and search strategy

Two researchers independently searched the PubMed, ISI Web of Science and MEDLINE databases from inception to February 2022 for related published studies. The literature search was limited to the English language. Index terms we used to search the indicate databases were ((lymphangioma) OR (lymphatic malformations) OR (LM) OR (LMs) OR (angiolymphoid)) AND (bleomycin). Secondary references included in these literatures were also recruited. If more than one paper was published on the same cohort, only the study with the largest sample size was included. Inclusion and exclusion criteria were shown in Table 1.

**Table 1:** Inclusion and exclusion criteria.

**Inclusion criteria**	(1) Evaluation of the efficacy of bleomycin on LMs.
	(2) Using descriptive study, case control study, cohort study, or randomized clinical trialdesign.
	(3) Researches had definite outcome indicators
	(4) Containing complete data information.
**Exclusion criteria**	(1) Not published in English.
	(2) Bleomycin combined surgery with for the treatment of LMs
	(3) Evaluation of efficacy between LMs and other sclerotherapy.
	(4) Studies of mechanisms based on genes or proteins.
	(5) Case reports, posters, guidelines, reviews, letters, and meeting abstracts.

### Data extraction

**Figure 1:**
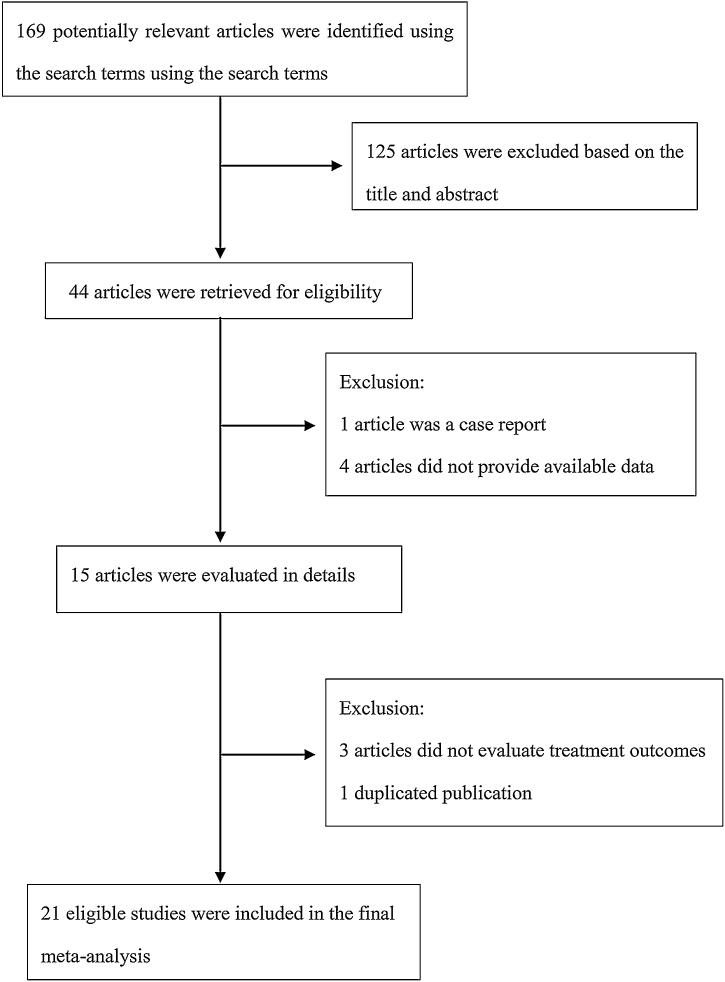
Flowchart of inclusion and exclusion of studies in the meta-analysis.

The following information was extracted from each study: 1) Name of the first author; 2) Year of publication; 3) Country where study was done; 4) Sample size of the study; 5) Age range of the study population; 6) Number of males and females; 7) Number of cases with effective treatment; 8) The dosage of used. If there was discordance among the 2 independent researchers for one study, its eligibility was decided by the 3rd investigator. 21 publications^14^, ^15^, ^16^, ^17^, ^18^, ^19^, ^20^, ^21^, ^22^, ^23^, ^24^, ^25^, ^26^, ^27^, ^28^, ^29^, ^30^, ^31^, ^32^, ^33^, ^34^ with 428 patients were comprised. Detailed information about flowchart of the study selection process was shown in Fig. 1.

### Quality assessment

The quality of each study was assessed according to MINORS (methodological index for non-randomized studies),^35^ which is a validated scale for non-randomized controlled intervention study.

### Statistical analysis

Fixed^36^ or random^37^ effects model, based on whether there was heterogeneity among studies. Heterogeneity was assessed by the Q-test and the I^2^ statistic.^38^ The random effects model was used when I^2^ value was greater than 50%.^39^ Subgroup analyses were performed by study design. Sensitivity analysis was performed to further explore the source of heterogeneity. Publication bias was assessed by Begg’s^40^ test and Egger’s^41^ test. All the statistical analyses were conducted using STATA version 14 (StataCorp LP, College Station, TX, USA).

## Results

### Study characteristics

A total of 169 papers identified through the database searches. Only 44 publications were potentially eligible after screening the titles or abstracts. Among them, 4 papers were excluded as they did not provide available data, and one was excluded as a case report. In addition, one duplicated publication was excluded and 3 were excluded as they did not provide evaluate treatment outcomes. Finally, we included 21 associated studies in the current meta-analysis (Table 2).

**Table 2:** Characteristics of studies include in the meta-analysis of the association between bleomycin and lymphangiomas.

Study	Year	Country	Study design	Sample size	Gender (M/F)	Age	Effective	Define	Dose
Vijai et al.^14^	2019	India	Retrospective study	21	8/13	3m‒18Y	19	Not available	0.5 mg/kg
Porwal et al.^15^	2017	India	Prospective study	8	5/3	4‒54Y	7	Macrocystic > 1 cm	0.5 mg/kg
								Microcystic < 1 cm	
Sindel et al.^16^	2017	Turkey	Prospective study	11	5/6	18‒51Y	11	Not available	15 mg
Ankur et al.^17^	2017	India	Retrospective study	27	15/12	3M‒52Y	17	Macrocystic > 1 cm	0.5 mg/kg
								Microcystic < 1 cm	
Ros et al.^18^	2017	Switzerland	Prospective study	16	5/11	1‒47Y	14	Not available	0.5 mk/kg
Lee et al.^19^	2017	Korea	Retrospective study	9	4/5	10‒67Y	7	Not available	1 mg/kg
Yılmaz et al.^20^	2017	Turkey	Retrospective study	10	6/4	2D‒32Y	10	Macrocystic > 2 cm	1 mg/kg
								Microcystic < 2 cm	
Raichura et al.^21^	2017	Mexico	Prospective study	13	5/8	1‒32Y	12	Not available	0.5 mg/kg
Ardıçlı et al.^22^	2015	Turkey	Retrospective study	13	Not available	1‒17Y	12	Macrocystic > 2 cm	0.25 mg/kg
								Microcystic < 2 cm	
Olimpio et al.^23^	2014	Brazil	Retrospective study	4	1/3	Not available	3	Macrocystic > 1 cm	0.5 mg/kg
								Microcystic < 1 cm	
Chaudry et al.^24^	2014	America	Retrospective study	31	10/21	3M‒31Y	12	Not available	1‒15 mg
Erikçi et al.^25^	2013	Turkey	Retrospective study	14	Not available	0‒9Y	12	Macrocystic > 1 cm	1 mg/kg
								Microcystic < 1 cm	
Kumar et al.^26^	2012	India	prospective study	35	Not available	1‒2Y	33	Not available	0.5 mg/kg
Harjai et al.^27^	2012	India	Retrospective study	30	Not available	0‒20Y	22	Not available	0.5‒1 mg/kg
Sandlas et al.^28^	2011	India	Prospective study	15	11/4	0‒12Y	13	Not available	0.6‒0.8 mg/kg
Niramis et al.^29^	2010	Thailand	Retrospective study	70	42/28	1M‒14Y	58	Not available	0.3‒0.6 mg/kg
Rawat et al.^30^	2006	India	Retrospective study	19	13/6	16D‒11Y	16	Not available	0.1‒0.5 mg/kg
Mathur et al.^31^	2005	India	Prospective study	10	7/3	2M‒10Y	7	Not available	1‒6 mg/kg
Sung et al.^32^	1995	Korea	Retrospective study	10	6/4	1Wks‒12Y	7	Not available	6 mg
Okada et al.^33^	1991	Japan	Retrospective study	29	13/16	1M‒12Y	25	Not available	1‒5 mg/cyst
Tanigawa et al.^34^	1987	Japan	Retrospective study	33	Not available	Not available	27	Not available	4.45 mg

### Results of meta-analysis

**Figure 2:**
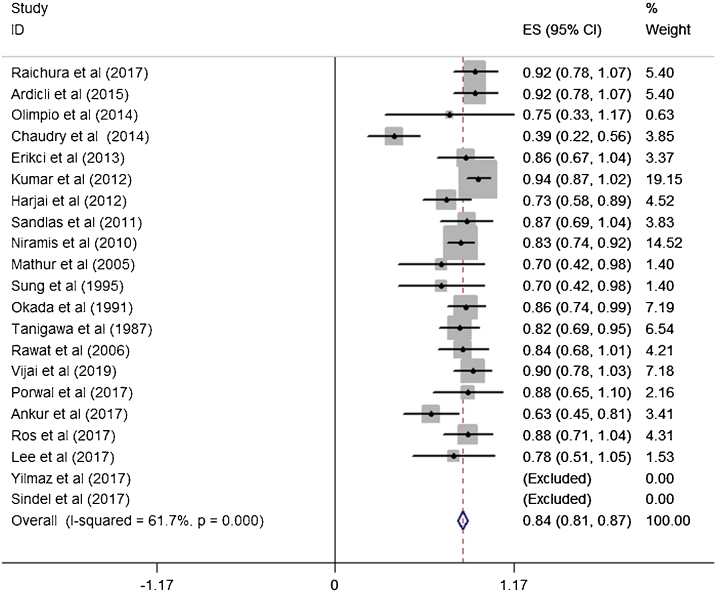
Forest plots of the summary effective rate with 95% CI for bleomycin sclerosing therapy.

A total of 21 studies (including 428 cases) were included in the meta-analysis of the efficacy after sclerotherapy. The results suggested that bleomycin was significantly effective in treating LMs. The combined effective rate was 84.0% (95% CI 0.81‒0.87) and ranged from 39% (95% CI 0.22‒0.56) to 94% (95% CI 0.87–1.02). Based on the meta-analyses, we also evaluated possible heterogeneity among the studies, and the heterogeneity found was substantial (I^2^ = 61.7%, *p* = 0.000) (Fig. 2).

### Subgroup analysis

**Figure 3:**
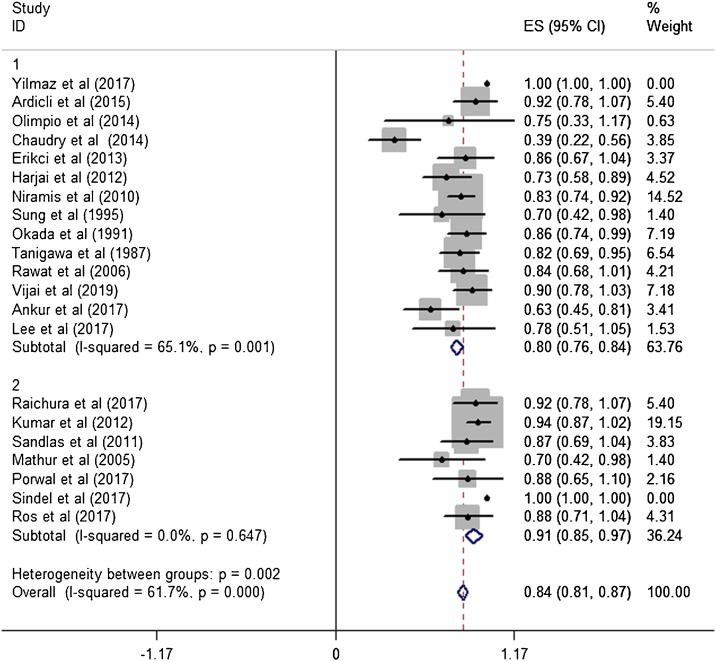
Effective rates with corresponding 95% CI in the prospective studies and retrospective studies.

**Figure 4:**
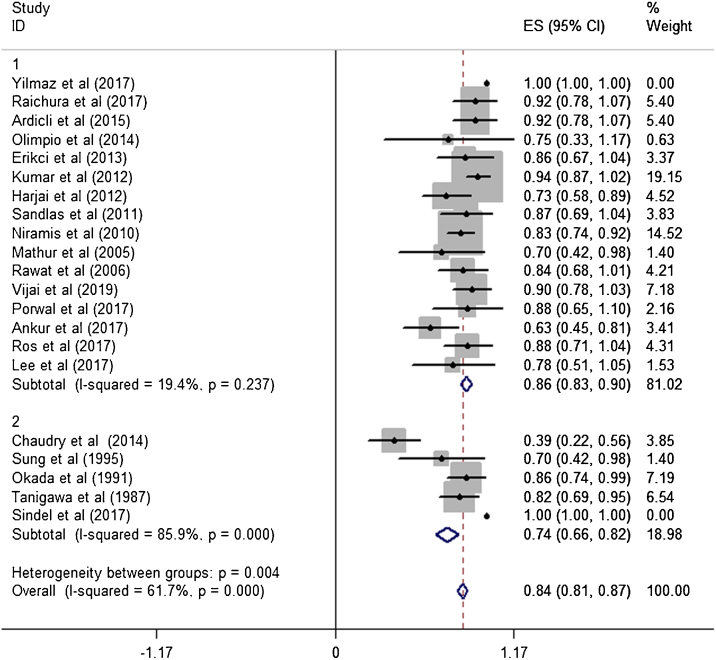
Effective rates with corresponding 95% CI in the weight-based group and the fixed-dose group.

The estimated effective rate was also analyzed by meta-analyses in subgroups according to the study design and dosage. The subgroups were divided into retrospective study group and prospective study group. It was observed that among retrospective study group (n = 310, study of Yilmaz et al. was automatically excluded from the system), the subtotal rate was 82% (95% CI 0.69‒0.95) and ranged from 39% (95% CI 0.22‒0.58) to 92.0% (95% CI 0.78–1.07). Among prospective study group (n = 97), the subtotal rate was 91.0% (95% CI 0.85‒0.97) and ranged from 70% (95% CI 0.42‒0.98) to 94.0% (95% CI 0.87–1.02) (Fig. 3). In terms of dosage, the association was significant in fixed-dose administration (subtotal rate was 74% [95% CI 0.66‒0.82], I^2^ = 85.9%, *p* = 0.000), but the heterogeneity of administration by weight was mild (I^2^ = 19.4%, *p* = 0.237) (Fig. 4).

#### Potential publication bias

**Figure 5:**
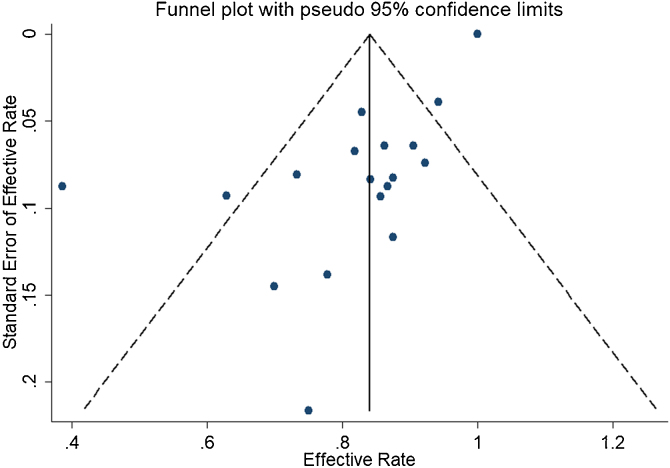
Funnel plots for detection of publication bias.

In the detection of publication bias, there was no significant publication bias in Egger's test (*p* = 0.059, 95% CI −3.81 to 0.082), but Begg's test did (*p* = 0.023). And the results also show that the funnel plot is asymmetric (Fig. 5).

## Discussion

The total of effective rate was 82%, which was almost consistent with previous literature reporting that bleomycin reduced symptoms by 84% in patients.^42^ At the same time, our study also revealed that the main effect of bleomycin was dosage. In the existing reports, there was no exact data to confirm the factors affecting the efficiency of bleomycin, so exploring the factors affecting the efficiency of bleomycin will become the focus of our next research.

As an anticancer drug, bleomycin has been proved to be effective against lymphoma, squamous cell cancer, testicular cancer, ovarian cancer, and other malignant tumors, but its incidence of toxic reactions and complications is high, especially pulmonary toxicity.^6^, ^43^ Meanwhile, Bennett et al. believed that the occurrence of chronic toxicity was correlated with the dosage of bleomycin and the age of patients. However, since its discovery in 1977 as a sclerotherapy for lymphatic deformities, bleomycin has become popular and even the most widely used sclerotherapy,^1^, ^42^ Yura et al. found that bleomycin was very effective in the treatment of LMs. In terms of side effects, in addition to fever caused by high doses of bleomycin, no serious complications such as leukopenia, rash, pulmonary fibrosis and growth inhibition were found.^1^ The main side effects include nausea, vomiting, skin discoloration, anaphylaxis and fever. The rare toxicities include interstitial pneumonitis, acute respiratory distress syndrome and pulmonary fibrosis, which may lead to heart failure. The short-term side effects usually associated with a single dose of serious, long-term side effects commonly associated with cumulative dose.^44^ Some studies used short Form 36 (SF-36) and patience-perceived change in health status (Global Rating of change scales) to investigate the long-term effect of treating LMs with bleomycin. The results showed that patients' symptoms and pain were improved, regardless of the size of position type.^42^

## Conclusion

In conclusion, the current meta-analysis suggested that bleomycin was highly effective in treating LMs, which should be widely applied to clinical treatment. And to some extent, the usage and dosage will affect the efficiency of bleomycin.

## Funding

Science and Technology Program of Jinan Municipal Health Commission (2022-2-144). Clinical Medical Science and Technology Innovation Program of JiNan science & Technology Bureau (202134070).

## Conflicts of interest

The authors declare no conflicts of interest.
